# Misregulation of the proline rich homeodomain (PRH/HHEX) protein in cancer cells and its consequences for tumour growth and invasion

**DOI:** 10.1186/s13578-016-0077-7

**Published:** 2016-02-13

**Authors:** Kevin Gaston, Maria-Angela Tsitsilianos, Kerry Wadey, Padma-Sheela Jayaraman

**Affiliations:** School of Biochemistry, University Walk, University of Bristol, Bristol, BS8 1TD UK; Division of Immunity and Infection, School of Medicine, University of Birmingham, Edgbaston, Birmingham, B15 2TT UK

**Keywords:** HHEX, PRH, Tumourigenesis, Cell proliferation, Cell migration, Cell invasion

## Abstract

The proline rich homeodomain protein (PRH), also known as haematopoietically expressed homeobox (HHEX), is an essential transcription factor in embryonic development and in the adult. The PRH protein forms oligomeric complexes that bind to tandemly repeated PRH recognition sequences within or at a distance from PRH-target genes and recruit a variety of PRH-interacting proteins. PRH can also bind to other transcription factors and co-regulate specific target genes either directly through DNA binding, or indirectly through effects on the activity of its partner proteins. In addition, like some other homeodomain proteins, PRH can regulate the translation of specific mRNAs. Altered PRH expression and altered PRH intracellular localisation, are associated with breast cancer, liver cancer and thyroid cancer and some subtypes of leukaemia. This is consistent with the involvement of multiple PRH-interacting proteins, including the oncoprotein c-Myc, translation initiation factor 4E (eIF4E), and the promyelocytic leukaemia protein (PML), in the control of cell proliferation and cell survival. Similarly, multiple PRH target genes, including the genes encoding vascular endothelial growth factor (VEGF), VEGF receptors, Endoglin, and Goosecoid, are known to be important in the control of cell proliferation and cell survival and/or the regulation of cell migration and invasion. In this review, we summarise the evidence that implicates PRH in tumourigenesis and we review the data that suggests PRH levels could be useful in cancer prognosis and in the choice of treatment options.

## Background

The proline rich homeodomain protein/haematopoietically expressed homeobox (PRH/HHEX), is a transcription factor encoded by the *HHEX* gene [1–3]. Although PRH was first identified in haematopoietic cells and it plays an important role in haematopoietic cell differentiation, the protein is expressed in a wide range of cell types in the embryo and the adult. During embryogenesis PRH is required for the development of multiple organ systems (including the forebrain, heart, liver, thyroid and thymus); it is also required at earlier points in embryonic development for the generation of the anteroposterior axis [4–19]. Thus, PRH knockout mice have a wide variety of defects including defective forebrain formation, liver formation, vasculogenesis and haematopoiesis and they are unable to survive gestation [4–6]. Analysis of RNA and protein in the adult indicates that PRH is widely expressed in many tissues including the haematopoietic compartment where it is preferentially expressed in myeloid cells but absent in T-lymphocytes. The importance of PRH in myeloid cells and leukaemia has been reviewed in detail previously [20] and recent studies implicate PRH in a number of other disease states including diabetes [21, 22]. Here we focus on the role of PRH in tumourigenesis and tumour cell biology.

### PRH structure

**Fig. 1:**
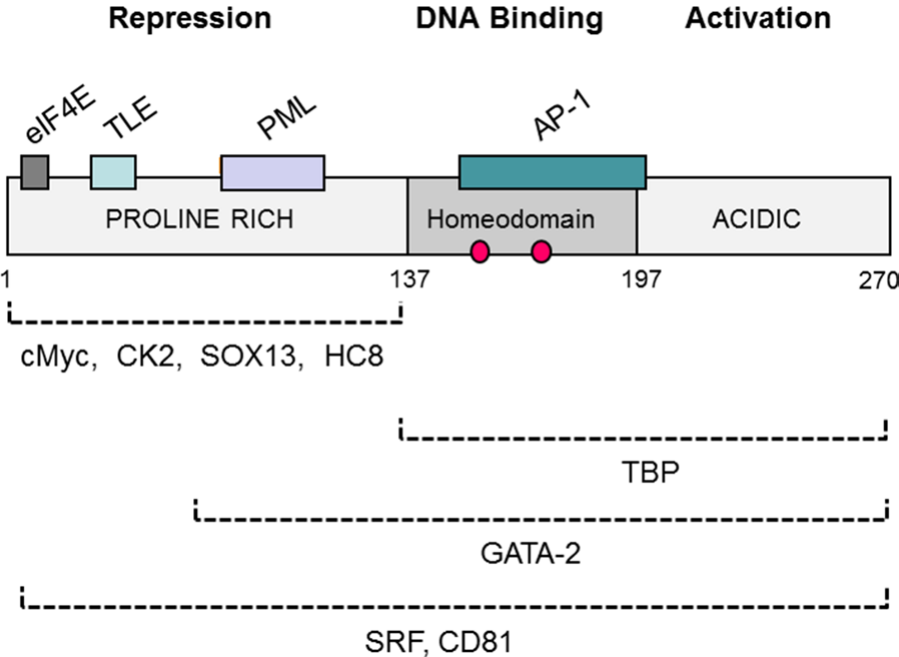
The PRH/HHEX protein and its interacting proteins. A diagrammatic representation of the human PRH protein. The PRH protein has three functional domains. The *boxed* and *bracketed areas* represent the regions of PRH that interact with the proteins indicated. The *brackets* indicate poorly mapped interactions. The *filled circles* represent residues that are phosphorylated by CK2 [30, 46-48, 51, 52, 54, 66, 76]

PRH is a 270 amino acid protein encoded by the orphan homeobox *HHEX* gene located on human chromosome 10. The PRH protein has a predicted molecular mass of 30 kDa, but in vivo and in vitro PRH forms homo-oligomeric complexes that appear to be octameric and hexadecameric [23–25]. These complexes are highly stable in vitro resisting denaturation by temperature and chemical agents [26]. The PRH monomer has three functional domains: a 136 amino acid N-terminal glycine-, alanine- and proline-rich domain, a central 60 amino acid proline-rich homeodomain, and a 73 amino acid acidic C-terminal domain (Fig. 1). The N-terminal domain can repress transcription when tethered to a heterologous DNA-binding domain [27]. The homeodomain mediates sequence-specific DNA binding [1, 24]. The C-terminal domain is required for the transcriptional activation of the sodium-dependent bile acid co-transporter (NTCP) gene and is therefore likely to be required for the activation of transcription in other contexts [28, 29].

### PRH oligomerisation and DNA binding

The isolated PRH N-terminal domain is an SDS (sodium dodecyl sulphate)-resistant dimer that lacks extensive α-helical or β-sheet secondary structure [23]. The N-terminal domain interacts with the PRH homeodomain and this facilitates oligomerisation [23]. Oligomerisation of PRH in cells has been demonstrated by in vivo cross-linking and in vitro gel filtration chromatography and analytical ultracentrifugation experiments imply formation of octameric and hexadecameric species [23]. The isolated PRH homeodomain binds to short DNA motifs, typical of homeodomain binding sites, and a truncated protein, lacking the N-terminal domain, binds to DNA sequences with the consensus 5′ ^C^/_T_^A^/_T_ATTAA^A^/_G_ 3′ [1, 24]. In contrast, the full length PRH protein binds with high affinity and specificity to tandem arrays of sequences related to these motifs [24]. Several PRH target genes contain tandem arrays of consensus and non-consensus PRH binding sites [24]. The Goosecoid promoter for example contains an array of PRH binding sites and PRH binding to this DNA has been shown to induce DNA condensation suggestive of the formation of a nucleosome-like octameric complex with wrapped DNA [24, 25]. However, DNA condensation is not sufficient for the regulation of transcription by PRH [25]. PRH can also be recruited to target genes via protein–protein interactions with other DNA binding proteins.

### The regulation of PRH expression

Transcriptional regulation of the *Prh/HHEX* gene is required for precise temporal and spatial distribution of gene expression, particularly in the context of early development and attaining cell-type specificity. Several regulatory elements—either intronic or situated in the 5′ flanking region of the *Prh/HHEX* gene—have been identified, as well as putative or experimentally established regulatory proteins and signalling pathways. One such regulator is the Sp family of transcription factors: in MH_1_C_1_ rat hepatoma and K562 human erythroleukemia cells, Sp1 and Sp3 bind GC-rich regions within the 5′ flanking region of the *Prh/HHEX* gene and activate transcription [31]. These elements appear to be cell-type specific since they are not functional in all cell types [32]. Similarly, GATA-1, GATA-2 and c-Myb activate *Prh/HHEX* transcription via intronic haematopoietic cell-specific enhancer-like elements [33] and the Thyroid follicular cell-specific transcription factors TTF-1 and Pax8 also activate *Prh/HHEX* promoter activity in a cell type specific manner [34, 35]. Additionally PRH activates transcription at its own promoter in a positive regulatory feedback loop [34, 35]. Further examples of regulatory factors include HNF3β and GATA-4 which activate the *Prh/HHEX* promoter in liver-derived HepG2 cells [36]. It is likely that cancer-associated changes in transcription factor activity result in altered PRH expression. However, the precise nature of the misregulation is expected to vary in different cancer types. In early development, *Prh/HHEX* expression is modulated by Wnt/β-catenin, transforming growth factor β (TGFβ), fibroblast growth factor (FGF) and bone morphogenetic protein (BMP)-mediated signalling pathways [18, 37–40]. The impact of these signalling pathways on *Prh/HHEX* expression in tumour cells and the effects of cancer-associated changes in signalling are very poorly understood. Recent work has shown that elevated *Prh/HHEX* expression occurs in Early T cell progenitor acute lymphoblastic leukaemia (ETP-ALL) and that this gene is a direct target of the LMO2 oncoprotein [41, 42]. *Prh/HHEX* transcription is activated by a triad of factors LMO2/FLI1/ERG bound at an intronic enhancer [42] and, additionally, chromatin immunoprecipitation experiments showed enrichment of LMO2, LDB1, LYL1, and GATA3 at the promoter and enhancer suggesting specific occupancy by these factors [41].

### The regulation of transcription by PRH

Like several other transcription factors PRH can activate or repress gene expression depending on the target gene (Table 1). PRH-repressed genes include chordin (CHRD), Goosecoid (GSC), endothelial cell-specific molecule 1 (ESM1), vascular endothelial growth factor A (VEGFA), and thyroglobulin (TG) [10, 43–45]. As might be expected, PRH has a variety of interacting proteins many of which are transcription factors (Fig. 1). PRH can repress transcription of target genes via the recruitment of members of the Groucho/TLE family of co-repressor proteins. An engrailed homology 1 (Eh-1) motif (30—TPFYIEDILD—39) within the N-terminal PRH repression domain interacts with the TLE1 Q-domain and C-terminal WD repeat domain. Mutation of phenylalanine 32 to glutamic acid (PRH F32E) negates these contacts and inhibits repression by PRH [27, 46]. However, PRH can also repress transcription by competing with the TATA box binding protein (TBP) for binding to TATA box sequences and by interacting with other transcription activators. For example, PRH binds to GATA-2 and prevents GATA-2 from activating transcription of the KDR gene encoding VEGFR2 [47]. Similarly, PRH binds to Jun and inhibits Jun-dependent transcriptional activation of the basic fibroblast growth factor (bFGF) gene FGF2 [48].

**Table 1:** Direct PRH target genes associated with cancer

Target gene	Function and pathways	Regulation	Evidence	References
ENG (Endoglin/CD105)	TGFβ co-receptor: neoangiogenesis, cell invasion, metastasis, cell proliferation	Activated	ChIP, EMSA	[49]
ESM1 (ESM-1/Endocan)	Cell–cell contacts: neoangiogenesis, metastasis, cell proliferation	Repressed	ChIP, EMSA	[43]
GSC (Goosecoid)	Transcription factor: epithelial-mesechymal transition	Repressed	ChIP, EMSA	[10, 24]
KDR (FLK1/VEGFR2)	VEGF receptor: neoangiogenesis, cell invasion, metastasis, cell proliferation	Repressed	ChIP, EMSA	[44]
VEGFA (VEGF/VPF)	Growth factor: neoangiogenesis, cell invasion, metastasis, cell proliferation	Repressed	ChIP, EMSA	[44]
FLT1 (VEGFR1)	VEGF receptor: neoangiogenesis, cell invasion, metastasis, cell proliferation	Repressed	ChIP, EMSA	[44]

PRH activates transcription of multiple genes include the genes encoding the TGFβ co-receptor Endoglin (ENG) [49], the Na(+)-bile acid cotransporter (NTCP/SLC10A1) [28] and the L-type pyruvate kinase (L-PK/PKLR) [50]. PRH activates transcription through multiple mechanisms via direct binding to promoter regions, as in the case of the NTCP promoter [28], or through association with other transcription factors to act as a transcriptional co-activator. For example, PRH interacts with hepatocyte nuclear factor 1α (HNF-1α) to promote HNF-1α-dependent transcriptional activation of L-PK [50]. Similarly, the interaction of PRH and serum-response factor (SRF) results in the activation of smooth muscle α-actin transcription via increased binding of SRF to DNA [51].

### Post-transcriptional regulation of gene expression by PRH

PRH can repress the transport of specific mRNAs via interaction with translation initiation factor 4E (eIF4E), thus inhibiting the translation of specific proteins [52]. PRH binds to eIF-4E in promyelocytic leukaemia protein (PML) nuclear bodies, inhibiting eIF-4E-mediated nucleocytoplasmic transport of transcripts including cyclin D1 [52]. Ectopic expression of PRH can disrupt PML nuclear bodies and block eIF-4E-dependent transport of cyclin D1 mRNA resulting in reduced cyclin D1 protein expression in leukaemic cell lines [52].

### The regulation of PRH activity by protein kinase CK2

Protein kinase CK2 (previously casein kinase 2) is a ubiquitously expressed enzyme implicated in a diverse range of cellular functions and processes including cell cycle progression [53]. CK2 is a serine/threonine kinase with the consensus target sequence S/T–X–X–D/E/pS (where X indicates any non-basic amino acid). CK2 exists as a heterotetrameric holoenzyme consisting of two catalytic α subunits and two regulatory β subunits. The PRH N-terminal repression domain binds to the β subunit of CK2 and CK2 phosphorylates two residues (S163 and S177) within the PRH homeodomain [54]. Phosphorylation at these sites inhibits PRH DNA-binding in vitro and in cells, and blocks the transcriptional regulation of PRH target genes [54, 55]. Furthermore, incubation of K562 cells with the translation inhibitor anisomycin indicated that hypo-phosphorylated PRH is longer lived than hyper-phosphorylated pPRH, and that expression of the latter is prolonged by treatment with the proteasome inhibitors MG132 and Lactacystin [55]. Thus, it appears that phosphorylation of PRH by CK2 targets the protein for proteasome-mediated cleavage. Interestingly, one cleavage product is a stable truncated PRH protein that lacks the C-terminal domain (PRHΔC), and this appears to operate as a transdominant negative regulator of full-length PRH by sequestering TLE co-repressor proteins [55]. Expression of PRHΔC leads to increased expression of target genes repressed by PRH including FLT1 (VegfR1), suggesting that the presence of PRHΔC could antagonise the inhibitory effects of PRH on cell proliferation.

### PRH in haematopoiesis and leukaemia

PRH is required for haematopoietic cell differentiation [56] at multiple stages of the differentiation process, reviewed in [20, 57]. Studies using embryoid body differentiation and blastocyst complementation have demonstrated critical roles for PRH in the development of definitive haematopoietic stem cells (HSCs) and B-cells [58–61]. Conditional knockout mice (Mx-Cre and Vav-Cre) revealed broadly similar results namely that HHEX is not required for maintenance of adult HSCs and myeloid lineages either postnatally, or at later time points, but is essential for the commitment of multiple lymphoid lineages at the stage of the common lymphoid progenitor. Although there are some differences between the two models presumably because of differences in Cre induction and the timing of knockout, both models show decreased expression of cyclin D1 and effects on lymphopoiesis [62, 63]. This decrease in cyclin D1 expression with *HHEX* knockout is also observed in ES cell derived haematopoietc colonies [61]. Overexpression of cyclin D1 in progenitor cell populations after inducible knock out (Mx-Cre) of *HHEX* rescues the B-cell developmental potential of PRH-null lymphoid precursors. Thus, PRH appears to regulate early lymphoid development by increasing cyclin D1 expression [62, 63]. Interestingly under conditions of stress haematopoiesis, that is, after sub-lethal irradiation, deletion of *HHEX* results in an inability of bone marrow cells to contribute to all bone marrow lineages as well as alterations in the proportion of long term and short stem cells, increased proliferation in vivo of stem cells and progenitors, and defects in T-cell populations [63].

PRH is implicated in several subtypes of leukaemia. Confocal microscopy and Western blot analysis demonstrate a substantial decrease in PRH protein levels and decreased nuclear localization of PRH in 13 of 13 primary AML (French American British classification M4/M5) and seven of seven blast crisis CML (bcCML) specimens but not in 11 of 11 M1/M2 AML, seven of eight acute lymphoid leukemia (ALL) specimens, or two of two chronic-phase CML [64]. Over-expression of eIF-4E transforms rat embryo fibroblasts and increased levels of this protein have been found in AML M4/M5 subtypes [64]. PRH binds to eIF-4E disrupting eIF4E nuclear bodies and repressing mRNA transport of eIF-4E targets such as cyclin D1 mRNA; in U937 human leukemic cells this results in the inhibition of cell proliferation [52, 64]. Thus PRH is a post-transcriptional repressor of cyclin D1 protein expression in leukaemic cells. The positive regulation of cyclin D1 mRNA expression by PRH inferred from knock out mouse experiments [61–63] is in contrast to the negative regulation of cyclin D1 protein expression in leukaemic cell lines. This apparent contradiction may be related to the lineage/differentiation state of the transformed cells compared to untransformed progenitors in vivo or it may indicate a subtle mechanism for the fine-tuning of cyclin D1 expression by PRH.

In keeping with an inhibitory role of PRH on the proliferation and transformation of leukaemic cells of myeloid origin, [44, 64–67] PRH directly represses the transcription of multiple genes involved in VEGF-signalling in leukaemic K562 cells. PRH knockdown in K562 cells results in increased transcription of these genes and increased VEGF autocrine signalling leading to increased cell survival [44]. These genes are regulated by PRH in other cells types: in endothelial cells, PRH has been shown to repress VegfR2 transcription although, in this case, repression is via an interaction with the transcription factor GATA-2 [47]. The derepression of these genes following PRH down-regulation is likely to be important in tumourigenesis.

Perturbation of the subcellular localisation of endogenous PRH has been proposed to be involved in the development of acute promyelocytic leukaemia (APL). The PML tumour suppressor protein regulates cellular signalling pathways controlling cell proliferation, apoptosis and senescence; PML can also interact with and negatively regulate eIF4E [68]. Chromosomal rearrangements observed in APL produce fusion proteins between PML and retinoic acid receptor α (RARα) [69]. PRH interacts with PML independently of its interaction with eIF-4E, and PRH over-expression disrupts PML nuclear bodies [52, 66]. Since PRH interacts with PML as well as with PML-RARα [66], PML-RARa is likely to interfere with both endogenous PRH and PML activity, promoting leukaemogenesis [57].

More recently it has been demonstrated that PRH can function as an oncogene in AML. PRH mRNA expression has been noted to be elevated in microarray studies from a variety of human AML samples. Moreover high PRH mRNA expression correlates with poor survival [70]. Importantly, PRH knockout in a mouse model of AML where the AML is initiated by expression of a MLL-ENL fusion protein showed that PRH is required for the initiation and maintenance of the leukaemia and functions alongside HOXA9-Meis1 as a transforming oncoprotein [70]. PRH recruits Polycomb co-repressor complexes to bring about the repression of a set of genes with protein products including the cell cycle inhibitors p16-INK4 and p19-ARF. This repression is essential to maintain the leukaemic blasts [70]. Hence targeting PRH is a potential therapeutic approach for MLL-ENL dependent AML and may also be relevant for other AML where PRH mRNA expression is elevated.

Thus it appears that in some situations alteration of endogenous PRH protein levels or nuclear localisation can contribute to AML whereas in others oncogenic transformation leading to elevated PRH mRNA expression can result in AML. The cell type that is transformed, that is whether transformation occurs in a differentiated cell or a progenitor/stem cell might account for these apparent contradictions. The complexity of PRH involvement in AML is underscored by the finding that PRH is directly involved in the initiation of at least one AML without involvement of additional transforming proteins [65]. In this case a cytogenetic abnormality generated the fusion of nucleoporin 98 and PRH (Nup98-HHEX), where the N-terminal domain of PRH was substituted by that of Nup98, culminating in the emergence of a leukaemogenic gene expression profile [65]. Transplantation of murine bone marrow cells expressing Nup98-HHEX into transgenic mice resulted in acute leukaemia though with a latency period of 9 months, suggesting that the translocation is a pre-requisite for disease induction but not in itself sufficient for leukaemogenesis [65].

PRH can also act as an oncogene in a subtype of T-cell acute lymphoblastic leukaemia known as early T-cell precursor-like ALL (ETP-ALL). PRH can phenocopy the Lmo2 oncoprotein in inducing self-renewal when overexpressed in mice and elevated PRH causes a T-cell leukemia, which is strikingly similar to that caused by Lmo2. These results suggested that PRH is an important mediator of Lmo2-driven T-cell self-renewal and leukemia [41, 67, 71]. Moreover Lmo2-transgenic mice with conditional deletion of *Prh/HHEX* showed a significantly delayed onset of the T-cell leukemia [41]. However, recent work showed that deletion of *Prh/HHEX* does not always block Lmo2-induced leukemia indicating that these proteins can act via parallel pathways [72]. Interestingly, deletion of PRH in the thymus of Lmo2 transgenic mice did result in a reduction in the transplantation capacity and the radioresistance of Lmo2-transgenic thymocytes but did not inhibit the development of the leukaemia [72].

### PRH in cancer

Down-regulation of PRH protein expression and/or aberrant subcellular localisation of PRH are associated with liver, breast, and thyroid tumours [73–75]. PRH expression is lower in Grade III (poorly differentiated) hepatocellular carcinomas (HCC) compared to Grade II (well-differentiated) HCC [75]. Moreover, PRH over-expression inhibits tumour formation by an HHC cell line in nude mice [75]. More recently query of the ONCOMINE database for PRH expression in tumour samples showed that significantly lower PRH mRNA expression occurs in activated B-cell-like diffuse large B-cell lymphoma, diffuse large B-cell lymphoma, lung adenocarcinoma, thyroid gland papillary carcinoma, superficial bladder cancer and pancreatic carcinoma [76]. As mentioned above PRH subcellular localisation is implicated in tumourigenesis: PRH is present in the nucleus and cytoplasm in normal breast epithelial cells, but in ductal and lobular breast carcinomas, PRH is located predominantly in the cytoplasm [73]. Similarly, in normal thyroid tissues and adenomas, PRH protein is present in the nucleus and cytoplasm whereas in both differentiated and undifferentiated thyroid carcinomas PRH is only present in the cytoplasm [74]. These findings suggest that PRH function is compromised in multiple carcinomas by decreased PRH expression and/or aberrant intracellular localisation.

The mechanisms that lead to the down-regulation of PRH activity and the processes linking this to tumourigenesis are not well understood. However, down-regulation of PRH might result in the misregulation of eIF-4E and PML activity in some cells types as described above. Similarly, down-regulation of PRH could result increased VEGF signalling through derepression of VEGF signalling genes [44]. The importance of this in tumour growth is discussed in the following section. However, increased VEGF signalling might also increase cell proliferation in some cell types as shown in myeloid cells [44]. PRH can also alter TGFβ signalling via direct transcriptional regulation of the TGFβ co-receptor Endoglin [49]. The role of TGFβ in tumourigenesis is complex and varies depending on tumour type and stage. TGFβ signals through TGFβ type I receptor (TβRI) and TGFβ type II receptors (TβRII). The TβRI receptors possess a cytoplasmic domain that phosphorylates intracellular proteins including members of the Smad family of transcription factors. Endoglin is a TGFβ co-receptor expressed in multiple cell types including endothelial cells [77]. Endoglin is important in angiogenesis and tumour growth but it also regulates cellular proliferation. Since PRH activates transcription of Endoglin, down-regulation of PRH activity would be expected to decrease Endoglin levels resulting in altered TGFβ signalling and consequent increased cell proliferation. However, Endoglin can also act independently of the TGFβ signalling pathway and down-regulation of PRH could also impact on tumourigenesis via TGFβ-independent pathways.

PRH activity could also influence tumourigenesis via regulation of the oncoprotein c-Myc [76]. c-Myc is a transcription factor that promotes cell cycle progression and participates in the control of apoptosis, cell differentiation and angiogenesis. c-Myc is important in a variety of human cancers and the c-Myc gene is frequently amplified in tumour cells. One way that c-Myc regulates transcription is via the formation of DNA binding heterodimers with Max. The N-terminal domain of PRH directly interacts with the c-Myc and disrupts the formation of c-Myc-Max heterodimers [76]. This diminishes the cellular activity of the c-Myc resulting in decreased cell proliferation [76]. Interestingly, eIF-4E and v-myc cooperate to transform primary rodent fibroblasts [78] suggesting that down-regulation of PRH could transform cells via misregulation of these two targets [76].

### PRH and cancer cell migration and invasion

Metastatic secondary tumours formed by disseminated cancer cells are responsible for the vast majority of cancer-related deaths. Metastasis requires a complex series of events leading from the initiation of cell migration and invasion into local tissues, entry of cells into vessel (intravasation), exit from vessels (extravasation), to the formation of micro- and ultimately macro-metastases. This is facilitated by a change in cell morphology and behaviour known as epithelial-mesenchymal transition (EMT), which involves simultaneous loss of epithelial cell features, such as E-cadherin expression, and the acquisition of mesenchymal properties, including protease secretion and increased cell mobility/invasiveness. PRH regulates several genes that are important in EMT, cell migration and cell invasion, including VEGF signalling genes, Goosecoid, Endoglin, and ESM-1 [10, 24, 43, 44]. Tumour-related neo-angiogenesis induced by VEGF signalling is pivotal for tumour growth as it provides nutrients and oxygen whilst eliminating waste products. Any upregulation of VEGF signalling brought about by decreased PRH activity would be expected to increase tumour growth. We and others have shown that PRH directly represses transcription of Goosecoid, a known inducer of EMT in breast tumour cells [10, 24, 79]. In addition, PRH activates transcription of Endoglin, a TGFβ co-receptor that can down-regulate TGFβ-dependent cellular responses [49]. Since TGFβ can induce EMT in some cells, the activation of Endoglin transcription by PRH would be expected to inhibit EMT. Recent work is consistent with this hypothesis since the activation of Endoglin expression by PRH inhibits the migration of prostate and breast cancer cells and the invasion of extracellular matrix [49]. ESM-1 is a dermatan sulfate proteoglycan and increased serum levels of this protein are associated with highly vascularized metastatic tumours and a poor prognosis in lung cancer and kidney cancer [80]. PRH directly represses ESM-1 transcription suggesting that the disruption of PRH activity is likely to be important for ESM-1-related neoangiogenesis [43].

### Altered regulation of PRH by CK2

Aberrant CK2 expression or activity is thought to be oncogenic in non-small cell lung cancer [81], head and neck cancer [82], prostate cancer [83], breast cancer [84, 85] and kidney cancer [86]. CK2 also exhibits oncogenic co-operativity with c-Myc and Ha-Ras, enhancing cell transformation [87–89]. CK2-mediated abrogation of tumour suppressor activity has been demonstrated to play a significant role in tumourigenesis. Phosphorylation by CK2 inactivates the tumour suppressor proteins, PML, connexin, and phosphatase and tensin homology protein (PTEN) [90]. For example, phosphorylation of PML S517 by CK2 facilitates proteasome-dependent proteolysis of PML and substitution of S517 with non-phosphorylatable alanine or pharmacological inhibition of CK2 inhibits tumourigenesis in vivo by increasing PML protein levels [90]. Interestingly, phosphorylation of PRH S163 and S177 by CK2 results in increased cleavage of PRH by the proteasome, and the consequent misregulation of PRH target genes [55]. Moreover, inhibition of CK2 or substitution of S163 and S177 with non-phosphorylatable cysteine residues restores PRH function. This suggests that CK2 inhibitors could restore PRH function in disease states that show down-regulation of PRH activity such as breast cancer, liver cancer and thyroid cancer and thereby restore normal cell functions. Similarly, kinases that regulate CK2 could be targeted in order to inhibit CK2 and thereby restore PRH function. Our recent work has shown that the BCR-ABL/Src kinase inhibitor Dasatinib decreases CK2 activity and PRH phosphorylation resulting in increased PRH-dependent repression of Vegf and Vegfr-1 in leukaemic cells [91]. Since Src activity is increased in many cancer types resulting in increased CK2 activity, Dasatinib could restore PRH function in several disease states.

## Concluding remarks

The importance of PRH in tumourigenesis and tumour progression is beginning to be more widely appreciated. Altered PRH levels and altered PRH subcellular localisation are associated with several cancers and with some subtypes of leukaemia. Moreover, PRH inhibits the ability of normal epithelial cells to proliferate, migrate and invade surrounding tissue implying that the loss of PRH will be important in multiple disease states. These data suggest that PRH could be a useful biomarker in cancer diagnosis and/or prognosis and an important target in some forms of leukaemia. Importantly, PRH is required for the sensitivity of some leukaemic cells to Dasatinib, an indirect inhibitor of CK2 activity, and is important in the response of these cells to the direct inhibition of CK2. This suggests that PRH levels and/or pPRH levels may be of value in the choice of cancer treatment options in a variety of contexts. However, PRH is post-translationally modified in cells as well as being cleaved by the proteasome. Some of the modified and processed forms of PRH have altered regulatory activities and thus a variety of PRH proteins are present in cells with potentially contrasting activities. Stringently characterised antibodies generated to recognise different forms of PRH are therefore urgently required as well as clinical studies that evaluate the value of the different forms of PRH in cancer diagnosis and prognosis.
